# Flange-Based Hand-Eye Calibration Using a 3D Camera With High Resolution, Accuracy, and Frame Rate

**DOI:** 10.3389/frobt.2020.00065

**Published:** 2020-05-29

**Authors:** Fang Wan, Chaoyang Song

**Affiliations:** ^1^AncoraSpring, Inc. and SUSTech Institute of Robotics, Southern University of Science and Technology, Shenzhen, China; ^2^Department of Mechanical and Energy Engineering, Southern University of Science and Technology, Shenzhen, China

**Keywords:** 3D scanner, hand-eye calibration, robustness, flange-based calibration, photoneo

## Abstract

Point cloud data provides three-dimensional (3D) measurement of the geometric details in the physical world, which relies heavily on the quality of the machine vision system. In this paper, we explore the potentials of a 3D scanner of high quality (15 million points per second), accuracy (up to 0.150 mm), and frame rate (up to 20 FPS) during static and dynamic measurements of the robot flange for direct hand-eye calibration and trajectory error tracking. With the availability of high-quality point cloud data, we can exploit the standardized geometric features on the robot flange for 3D measurement, which are directly accessible for hand-eye calibration problems. In the meanwhile, we tested the proposed flange-based calibration methods in a dynamic setting to capture point cloud data in a high frame rate. We found that our proposed method works robustly even in dynamic environments, enabling a versatile hand-eye calibration during motion. Furthermore, capturing high-quality point cloud data in real-time opens new doors for the use of 3D scanners, capable of detecting sensitive anomalies of refined details even in motion trajectories. Codes and sample data of this calibration method is provided at Github (https://github.com/ancorasir/flange_handeye_calibration).

## 1. Introduction

Growing adoption of high-fidelity 3D scanners provides a versatile sensing solution for novel robotic systems to perceive the unstructured, yet dynamic, physical environment with refined details in geometry, color, texture, and insights (Weingarten et al., 2004; Qi et al., 2017; Wang et al., 2019). There is an engineering trade-off during the design and development of 3D scanners in the measurement area, accuracy, and frame rate (Sarbolandi et al., 2015). When interacting with the robotic manipulators, it is generally accepted that the capability of sensing high-quality 3D motions in real-time would contribute to the overall advancement of robotic research and applications (Kagami et al., 2002; Kanade, 2012). However, it remains unclear about the potential impact due to the limited research, which motivates this paper.

Machine vision contributes to the robot system by providing the geometry data of the physical world as well as the robot itself. The hand-eye calibration is a geometric synchronization process among the camera, robot, and environment in the spatial domain (Shah et al., 2012). Classical vision system usually requires an external calibration object of high precision to be used as the geometric baseline, which exhibits a standardized geometry feature for optical measurement and algorithmic processing (Tsai and Lenz, 1989; Kahn et al., 2014; Yang et al., 2018). In general, it is an involved and expensive process which requires the high-quality manufacturing of the calibration object, the proper assembly of the robotic vision system for calibration (Karabegovic et al., 2006), and advanced feature engineering algorithm for calculating the calibration accuracy (Heikkila, 2000). Besides machine vision, there are also tactile-based sensing solutions for object recognition (Chin et al., 2019; Yang et al., 2020b).

**Figure 1:**
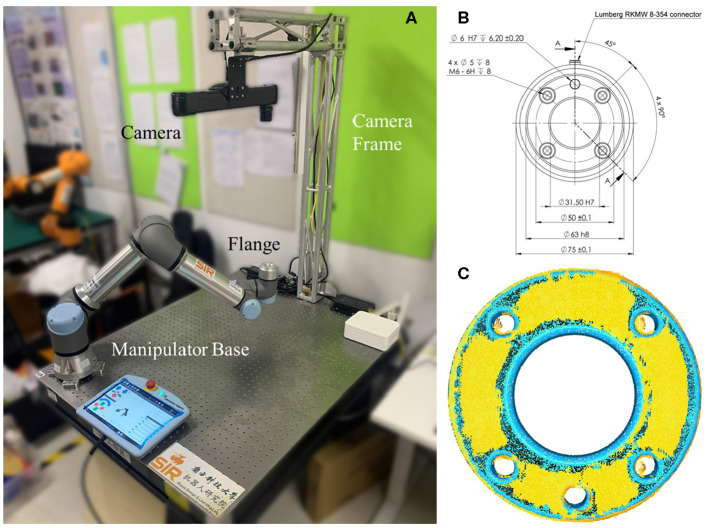
The proposed flange-based hand-eye calibration method, including **(A)** the experiment setup of a minimum robot system the involves a UR5 e-series and a Photoneo MotionCam-3D camera, both installed on a vibration isolation table; **(B)** the mechanical drawing of UR5 e-series robot flange that follows ISO 9409-1-50-4-M6; and **(C)** an illustration of the hand-eye calibration results using the proposed method with accuracy as high as the camera's, probing the hardware limit.

With the growing maturity in 3D depth-sensing technologies (Stoykova et al., 2007; Halmetschlager-Funek et al., 2019) and the international standardization of the robot design and manufacturing, it opens new doors through direct measurement and sensing of the depth data. For example, as shown in Figure 1, with the availability of high-quality 3D depth scanners (Zhang, 2018), one can potentially exploit the existing geometric features on the robotic manipulator under internationally recognized standardization, i.e., International Standard Organization (ISO) 9409-1:2004, for a direct measurement and calibration ISO (2004). We are interested in exploiting such geometric features using depth sensors through particular a dynamic measurement of high resolution, accuracy, and frame rate, which is the focus of this paper.

### 1.1. Related Work

Technical specifications, including resolution, accuracy, and frame rate, contribute positively to the quality metrics of a 3D scanner. For example, line laser scanners is a commonly adopted in engineering automation where a laser is modified to project a line over the target object for high-resolution, continuous measurement (Gestel et al., 2009). Such line laser profilers are usually limited to conveying systems due to the underlying profiling process in a line-by-line manner. Stereo vision systems is another classical method that mimics the triangulation principals of binocular vision (Aguilar et al., 1996). It can easily cover a much wider area per frame rate but usually limited in resolution and accuracy for objects with fewer features. Time-of-flight (ToF) technology measures the traveling time of light emitted by illumination to an object and back to a detector (Kolb et al., 2010; Foix et al., 2011). Structured light measures the deformation of certain patterns of particular design over the target area (Salvi et al., 2010). While the ToF cameras are usually developed for consumer usage, which can produce point clouds in real-time frame rate with compromises in accuracy and resolution, structured light cameras exhibit the opposite characteristics for engineering automation. The 3D perception technologies have been widely applied to high-accuracy reconstruction (Chen et al., 2019), defect and surface inspection (Tang et al., 2019), and intelligent robot (Wan et al., 2020; Yang et al., 2020a).

**Figure 2:**
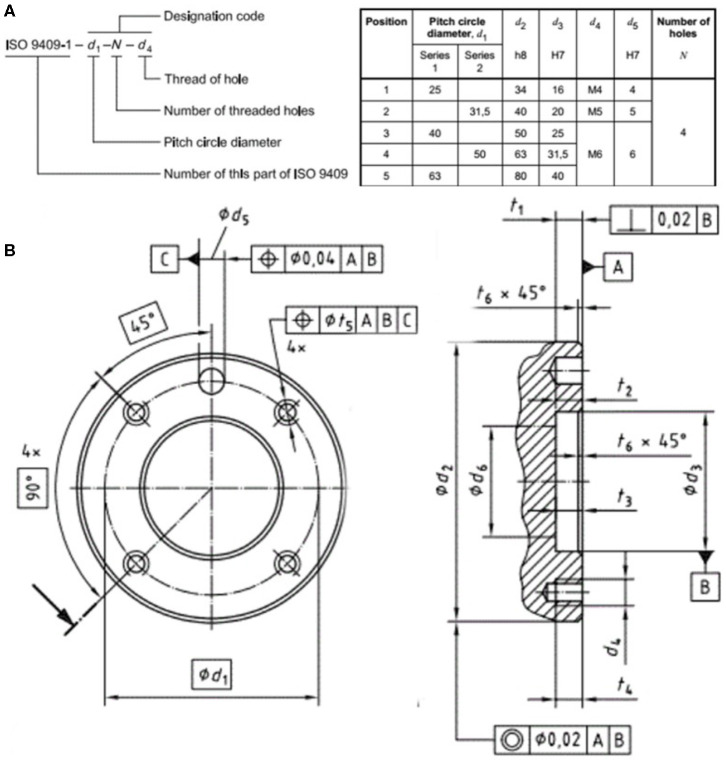
Except from the ISO 9409-1: 2004 on the design standardization of robot flanges, including **(A)** the designation code format and recommended dimensions, and **(B)** the detailed mechanical drawings and notations.

The standardization of robot interfaces at various levels is of critical importance to the reusability and exchangeability of robot systems, including mechanical, electrical, and communication. Among the International Standard Organization's catalog 25.040.30 industrial robots and manipulators, the ISO 9404-1 specifies the design standardization of the mechanical interfaces or the fixture design on the tool flange in particular. Figure 2 is adapted from the latest version released in 2004, which specifies the critical mechanical interfaces including the threaded holes referencing circle diameter in *d*_1_, the flange's outer circle diameter in *d*_2_, the number of threaded holes *N* to be used for fixture, the size of the threaded holes *d*_4_, etc.

### 1.2. Proposed Method and Original Contributions

In this paper, we explore the potential use of a direct hand-eye calibration method utilizing the depth-sensing data of the robot flanges under ISO standardization. In particular, with the advanced capability of the depth-camera available, we further explored the potential use of our proposed method for hand-eye calibrations in a dynamic setting during motion. Furthermore, we found that the availability of such high-quality point cloud data in high frame rate enables us to extend our proposed method for a whole-body calibration tacking for the robot system. The following summarizes the original contributions of this paper:

- A novel hand-eye calibration method of high accuracy, utilizing the existing, standardized design features on the robot flange with reduced system error;
- A systematic exploration of a structured-light 3D scanner of high resolution, accuracy, and frame rate for robotic calibration and manipulation;
- A direct whole-body motion tracking and calibration monitoring using direct depth sensing in a dynamic setting of robotic manipulation;
- An exploratory investigation of the high-frame-rate data redundancy problems in depth sensing, which was not discussed in previous literature.

The rest of this paper is structured as follows. Section 2 formulates the hand-eye calibration problem with a direct 3D measurement of the robot geometry, reviews the standardized design features on the robot flange, and introduces the proposed calibration method. This section also introduces the technical features of the high-quality 3D scanner to be used in this paper. Section 3 presents the experiment results using flange-based calibration method in the common static setting, as well as the challenging dynamic setting. We also explored the potential use of 3D point cloud measurement for trajectory tracking and error detection. Section 4 presents the discussions of the proposed method and the research potentials of such high-quality 3D scanner. Section 5 concludes this paper.

## 2. Methods

### 2.1. Problem Formulation

The hand-eye calibration problem involves the kinematic synchronization of the spatial transformation among four coordinate systems, including the robot flange {*Flan*}, manipulator base {*Base*}, camera sensor {*Cam*}, and calibration marker {*Mark*}. In the example of an Eye-on-Base configuration, which is also called Eye-to-Hand configuration, a camera is installed on a structure where the relative positioning to the manipulator base is a fixed matrix ${}_{Base}^{Cam}Ĥ$. The calibration marker is usually fixed on the wrist joint near the robot flange, denoted as ${}_{Flan}^{Mark}Ĥ$. As a result, we can express the coordinate transformation of the Eye-on-Base configuration using Equation (1).

(1) $${}_{Flan}^{Base}H\;\cdot {\;}_{Mark}^{Flan}\hat{H}{=}_{Cam}^{Base}\hat{H}\;\cdot {\;}_{Mark}^{Cam}H$$

A common method to solve Equation (1) is by using iterative methods to solve a general equation of *AX* = *YB*, where $A{=}_{Flan}^{Base}H$ is a known matrix depending on the robot system's hardware specification. $B{=}_{Mark}^{Cam}H$ is a calculated matrix based on the camera's optical measurement of the object in forms of a 2D image or 3D point cloud, $X{=}_{Mark}^{Flan}Ĥ$ and $Y{=}_{Cam}^{Base}Ĥ$ are two unknown matrix to be solved.

Literature and engineering practices have suggested many different marker designs for calibration. As one might notice, solving Equation (1) is not an easy task due to the involvement of the {*Mark*} frame on the calibration object. Common designs of the markers for 3D camera take the shape of a sphere with certain texture specific to the technology of the vision system. The problem to be solved in this paper is to find a robust hand-eye calibration method without using any external marker.

### 2.2. Flange-Based Hand-Eye Calibration

Most robot manipulators are built with excellent repeatability through manufacturing and assembly but suffer from accuracy due to the inverse kinematic computation involved during control (Elatta et al., 2004). Other kinematic calibrations concern the spatial synchronization between the tool and the flange, or different robot systems in co-manipulation tasks (Wu et al., 2016). The calibration process usually involves a specially designed marker as the measurement baseline for accuracy. The literature also suggested potential techniques for object-less calibration, but most of them involve complex operations in algorithmic level (Li et al., 2018), which introduces further uncertainties in system errors.

In this paper, we propose a marker-less hand-eye calibration method for high-fidelity 3D scanners by utilizing standardized design features on the robot flange, namely flange-based hand-eye calibration method. By letting the Tool Center Point (TCP) of the tool flange be the marker point, we can obtain the coordinates of the TCP positions with reference to the robot base {pBasei∣i=1,2,...,n} by directly reading from the robot controller and obtain the coordinates of the same TCP positions with reference to the camera {pcami∣i=1,2,...,n} by finding the center of the circle features of the tool flange from point cloud data. The steps of identifying the TCP point by the camera are as follows. Firstly, the tool flange plane is identified by plane fitting using random sample consensus (RANSAC) after removal the irrelevant part of the point cloud, as shown in **Figure 9**. Then the outer circle of the tool flange is found using RANSAC fitting with known radius and the TCP is the center of the circle. According to Umeyama (1991), the least-squares estimation of transformation between the robot base and the camera ${}_{Cam}^{Base}Ĥ$ can be reformulated as following

(2) min1n‖CamBaseR . Campi+CamBaset−pBasei‖

where ${}_{Cam}^{Base}R$ and ${}_{Cam}^{Base}t$ are the rotation and translation between the robot base and the camera. Equation (2) has an analytical optimal solution using Singular Value Decomposition (SVD) method and a minimum of four non-coplanar points are required for calculation. The readers are encouraged to refer to the Github page for further implementation details.

### 2.3. 3D Camera With High Resolution, Accuracy, Frame Rate

**Figure 3:**
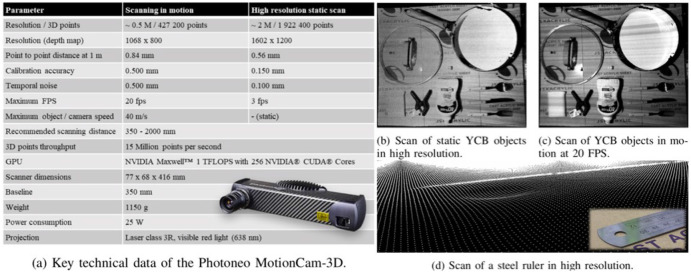
The MotionCam-3D camera from Photoneo, including **(a)** the technical data sheet; and the captured point cloud of YCB objects **(b)** in high resolution static scan with nearly 2 million points captured at up to 0.150*mm* accuracy under 3 FPS; and **(c)** in motion with nearly 0.5 million points captured at up to 0.500*mm* accuracy at the maximum 20 FPS; **(d)** the captured point cloud of a steel ruler with thickness of 1*mm*.

The successful implementation of the proposed method requires technical specifications from the depth sensors to capture the standardized design features on the robot flange. In this paper, we experiment with a depth-sensor from Photoneo, MotionCam-3D, to demonstrate the proposed method. As shown in the data sheet produced in Figure 3, MotionCam-3D is capable of producing high-quality point cloud data in a competitive resolution, accuracy, and frame rate. The MotionCam-3D is a structured light camera implemented by a custom CMOS image sensor. Capturing high-density point cloud in near real-time frame rate enables the camera to track movements as fast as 40 meters per second.

A practical problem with the MotionCam-3D is probably saving the data. At the top fps of 20 in the highest resolution of nearly 0.5 million points within 1,068 × 800 depth map, a total of nearly 10 millions points need to be processed every second, producing more than 400 MB per second data to be saved on a hard disk. This could be a challenging task on the computing hardware to process such amount of data in real-time, which requires efficient algorithm design or matching hardware to make use of the data. Such data redundancy is useful in inspection, measurement, or detection tasks to identify anomalies but unclear about its contribution to the improved manipulation of robots, which is one of the research questions to be explored in this paper.

## 3. Experiment Results

### 3.1. Experiment Setup

**Figure 4:**
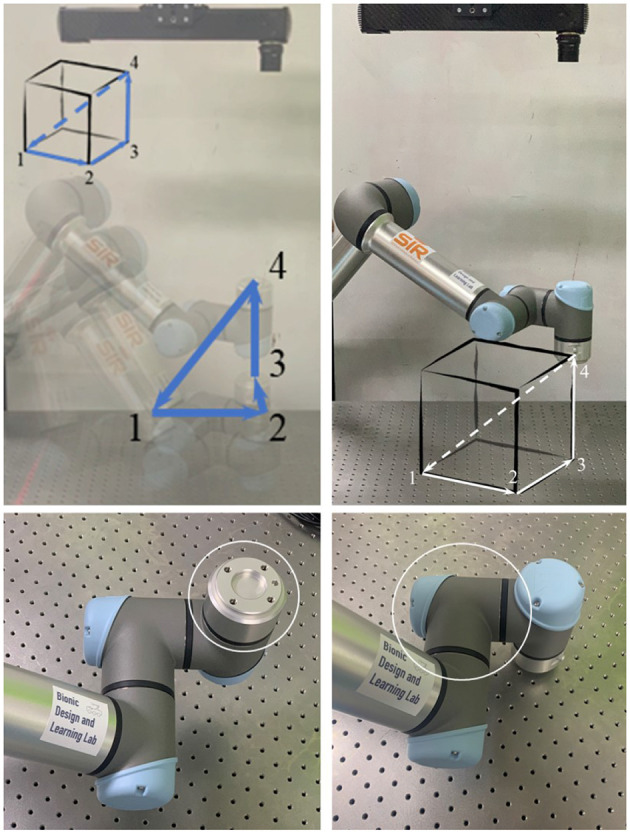
Experiment setup using a minimum robot system, where the robot flange is configured to move along four way points on the vertices of a cube.

The experiment is conducted using a minimum robot system with a vision sensor. As shown in Figure 4, a UR5 e-series by Universal Robot is fixed on one side of a vibration isolation table, whereas an L-shape structured built with aluminum truss modules are installed on the other side. The Photoneo MotionCam-3D is fixed on top of the aluminum truss facing downwards to the tabletop. Please note that no gripper was used in the experiments in this paper.

Although the proposed method is implemented by referencing to the robot flange, we are not bounded by such geometric constraint thanks to the high-quality point cloud captured by MotionCam-3D. We also demonstrate the extension of the proposed method, which applies to the general geometric features of the robot.

### 3.2. Flange-Based Calibration in Static and Motion Modes

Flange-based calibration was first conducted using point clouds captured in high-resolution, static mode. The robot moved in a grid of 4 × 4 points within a 200 × 200mm^2^ area in the *x*-*y* plane with a random vertical perturbation at around *z* = 220mm. The robot stopped at each grid point while the scanner took a picture and saved the point cloud. In total, 16 pairs of point cloud and the corresponding robot pose were collected. After obtaining the hand-eye matrix, the 6D calibration error matrix is estimated by projecting the mesh model of the tool flange in the camera coordinate with a particular robot pose for verification and computing the registration matrix between the mesh model and the captured point cloud using iterative closest point (ICP) algorithm (Rusinkiewicz and Levoy, 2001). The results of the calibration errors in static mode are reported in Table 1. The translation errors are about 1 mm in all three axis. The rotation errors can also lead to displacements when applying the hand eye matrix. For example, the 0.19 degree error in pitch can lead to 1.66 mm displacement at 0.5 meter away from the camera estimated by *R*× θ where *R* is the working distance from the robot tool to the camera and θ is the rotation angle in radian.

**Table 1:** The collected error compensation values for the flange-based hand-eye calibration methods in static and motion modes.

**Mode**	**Translation (mm)**			**Rotation (degree)**		
	**x**	**y**	**z**	**roll**	**pitch**	**yaw**
Static	1.57	−1.07	−1.12	−0.06	−0.19	0.09
Motion	18.89	−44.90	6.22	−4.76	−1.61	2.38

We conducted another experiment by capturing the point cloud of the robot flange at up to 20 frames per second (PFS). In this experiment, the robot moves along a trajectory by passing four vertices of a cubical space of 200 × 200 × 200mm^3^. The motion is programmed through the teach pendant attached to the robot controller using moveJ command. The results of the calibration errors in motion mode are also reported in Table 1. Note that due to the limited access to the timestamp data in Photoneo software, the point clouds for motion mode are not synchronized with the corresponding robot poses, which may cause the exaggerated differences between the static and motion modes in Table 1.

More importantly, the calibration errors can be compensated to the hand-eye matrix by right multiplying the hand-eye matrix computed from SVD method by the error matrix. After error compensation for the experiment values collected in Table 1, we were able to achieve a calibration accuracy as high as the camera's precision at 0.150 mm with respect to the verification point cloud. The calibration process essentially works like an initial guess followed by the ICP error adjustment.

### 3.3. Flange-Based Motion Tracking

**Figure 5:**
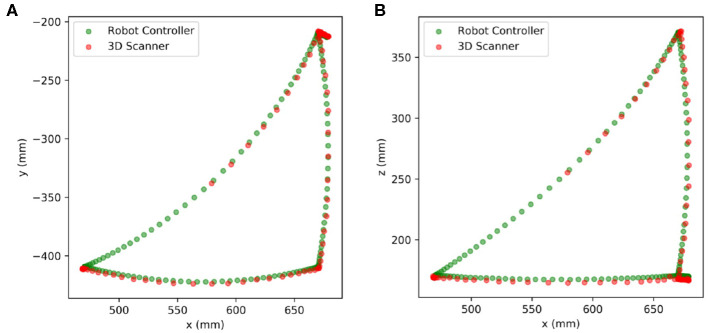
Trajectory tracking of the TCP center with moveJ. **(A)** In *x-y* plane **(B)** In *x-z* plane.

In this section, we present an extension of the proposed method for trajectory tracking by utilizing the high-frame-rate motion scanning capability of the MotionCam-3D camera. After calibration, we could perform a continuous detection of the robot flange while the robot is in moving. We used the same four way points as the previous experiment and tracked the TCP trajectory on robot flange in 20 FPS. The results are plotted in Figure 5, which includes the position data collected from the robot controller in green dots against those detected by the 3D scanner in red dots. The results in Figure 5 demonstrated the versatility of the proposed method with the availability of quality point cloud data collected in high frame rate.

**Figure 6:**
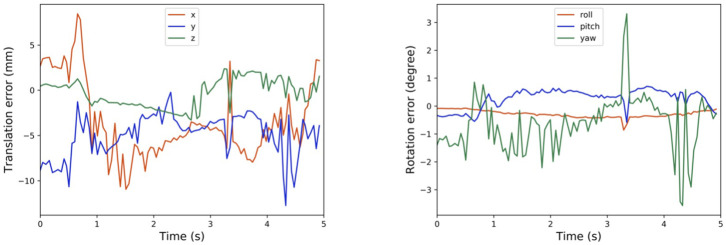
Tracking calibration error using robot flange.

Besides, we can also plot the history of the calibration errors during motion tracking of the robot flange in Figure 6. There is a small delay of about 140ms between the timestamp of the on-board image capture and that of received by the computer, which is synchronized later manually when plotting Figure 6. It seems that, from the collected data, the translational components of the calibration error can be greater than −10mm at some point in Figure 6. However, the rotational components remain reasonably small, which complies with general observations. This relatively exaggerated calibration error could be caused by the imprecise synchronization of timestamps between the point cloud collected from the camera, and the robot poses read from the controller.

### 3.4. Tracking of the Calibration Error

A limitation of the proposed method is the geometric features on the robot flange, which may be obscured by the end-effector during normal operation of the robot system, causing unnecessary troubles when using the proposed method. In this section, we demonstrate an extension of the proposed method by using other parts of the manipulator for trajectory tracking using quality point cloud data collected in high frame rate.

**Figure 7:**
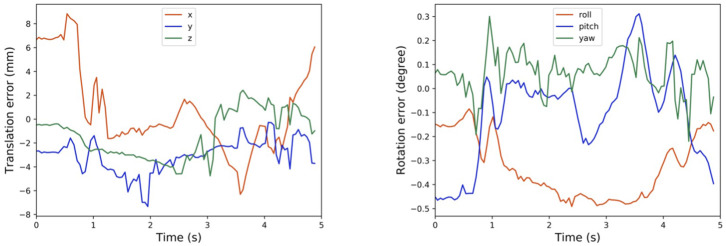
Tracking calibration error using the first wrist link.

Instead of using the robot flange, we could alternatively use other parts of the robot for calibration measurement and trajectory tracking. For example, we have shown in Figure 7 the history of calibration errors during motion tracking of the first wrist link on the UR5 e-series. The CAD file of the link could be acquired from Universal Robot website. Moreover, the results in Figure 7 exhibit consistent behaviors as those reported in Figure 6. Alternatively, one can choose any part of the robot to perform the same calibration process, as long as the chosen part is convenient to be captured by the camera during motion.

**Figure 8:**
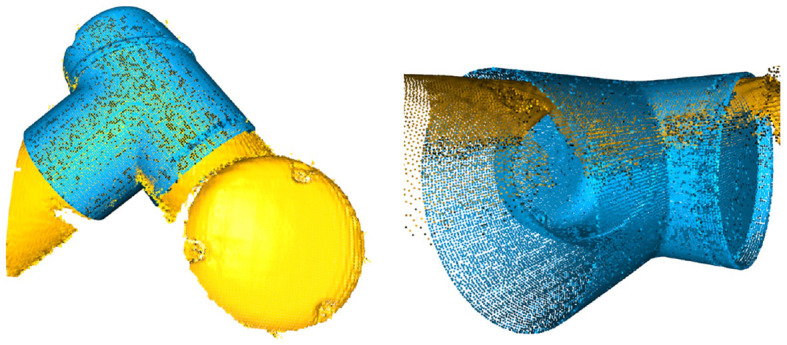
The point cloud overlay between those captured by the 3D scanner (yellow) and those generated from the CAD model (blue) of the wrist link 1 on a UR5 e-series, and note the mismatch of the point clouds on edge.

In cases where the CAD models are not directly available, one can exploit the MotionCam-3D camera to reconstruct a high-quality point cloud model of the target object for tracking. Then, following the same method as above, one can further extend the method using any other object for motion tracking, which demonstrates the versatility of the proposed method. Our current results suffer from the limited access to the timestamp synchronization between the captured point clouds and the robot poses, which could be the cause of the exaggerated calibration errors in the translational component in Figure 7. An illustration of the point cloud overlay is shown in Figure 8. It should be noticed that the collected error compensation values in Table 1 in static mode is much smaller than those collected in motion mode. Further understanding of MotionCam-3D's working principals may be necessary to evaluate the point cloud quality in motion, as well as its usage for dynamic scenarios for robotic manipulation.

## 4. Discussions

### 4.1. Point Cloud Quality in Static and Motion Modes

**Figure 9:**
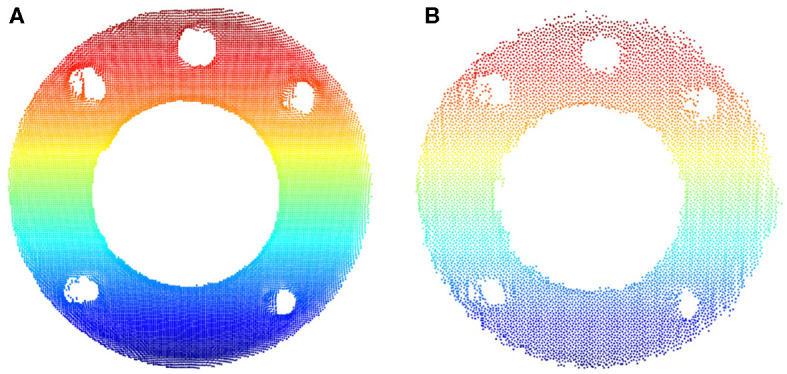
Comparison of the captured point clouds about the robot flange in different motion modes. **(A)** Static scan. **(B)** Scanned in motion.

The capability of capturing high-quality point clouds of the target object in static and motion modes is an important feature offered by the MotionCam-3D camera. Shown in Figure 9 is the comparison of the captured point clouds about the robot flange in static and motion modes. The static scan in Figure 9A produced a point cloud with higher density in general and fewer noises on the edges than the one scanned in motion shown in Figure 9B. For hand-eye calibration problems where the accuracy of the sensor is of greater importance, the static mode offers a refined measurement of the geometric details that enables direct use of 3D scanners for hand-eye calibration. However, in other scenarios such as trajectory monitoring and error detection, the point cloud of the robot flange generated from the motion mode offers a reasonable measurement of dynamics with visible details.

In general, a growing versatility in robotic manipulation research and applications are to be expected in the near future. Besides high-quality point cloud in different modes, other products such as the fourth edition of the Microsoft Kinect, i.e., Azure Kinect, offers a much-improved depth-sensing within a smaller form factor comparing to previous models with the addition of far-field speech and sound capture, and edge computing for AI.

### 4.2. Direct Hand-Eye Calibration in 3D

In the classical hand-eye calibration process, it is difficult to quantify the calibration error accurately because the ground true transformation is not known. Li et al. (2018) used the calibration results using a chessboard pattern as the benchmark to evaluate calibration methods without calibration object, achieving an error about 2 mm. Wu et al. (2016) proposed a simultaneous hand-eye calibration method using reflective marker and the experiment with an NDI Polaris optical tracker (RMS repeatability 0.1 mm) achieved translational errors of 2 mm and rotational errors of 0.5 degree. With the availability of high-accuracy 3D scanners, our method without calibration object achieves translational errors of 1 mm and rotational errors of 0.1 degree with much less data and computation. Furthermore, after error compensation, we are able to push the calibration accuracy to reach the hardware limit at 0.15 mm, namely the precision of the 3D scanner.

The standardization in the design and manufacturing of the robot flanges, or other similar features, offers a convenient and useful reference for hand-eye calibration. By using depth-sensors of reasonably high-quality, we experimentally verified a comparable calibration accuracy as high as the camera's, probing the hardware limit. The combination of robotic manipulators with depth-sensors has been widely adopted in fundamental research as well as applications such as bin-picking in e-commerce fulfillment, machine tending for intelligent manufacturing, palletizing and de-palletizing for logistics, and healthcare industry for collaborative inspection and measurement.

We conducted extensive experiments to verify the robustness of the proposed method with a range of robotic manipulators, including UR10 e-series, Franka Emika, UR5, and Aubo i5, with other industrial-grade depth sensors such as Photoneo Phoxi S model and M model, and consumer-grade ones such as Microsoft Azure Kinect DK. Detailed results of the experiments are reported on the Github page for the readers' interests.

### 4.3. Whole-Body Tracking of the Calibration Error

The high-frame-rate 3D measurement offered by the MotionCam-3D camera enables us to further experiment with our proposed method beyond the design constraint in the robot flange. As shown in the experiment results in Figure 7 of tracking the calibration error using the CAD model of the wrist link against the measured point cloud, one can quickly reproduce the method to any visible part of any robotic manipulator with ease. Even for the case where the manipulator or target object's CAD model is not available, one can exploit such high-frame-rate 3D motion scanning by reconstructing a high-quality point cloud model first in a short amount of time, and then achieve the same goal such as calibration error tracking, or pose detection and measurement in general, which opens the doors for future research and applications.

### 4.4. Trade-Offs With Data Redundancy

However, there remains a significant challenge in trade-offs with data redundancy. The influx of such point cloud data poses a significant challenge for embedded computation as well as real-time processing for responsive interaction with robotic hardware. Unless equipped with powerful hardware, such richness or even redundancy in point cloud data may not be suitable for real-time processing, but more applicable to the 3D measurement and detection tasks where an educated decision is to be calculated later. For most robotic manipulation tasks, the density and quality of the point cloud may not be the bottleneck of the problem, but the systematic integration with the target object, intended process, and the operating environment, which is worthy of discussion in the future research.

## 5. Conclusion

In this paper, we proposed a novel use of the depth sensors for a direct hand-eye calibration with a reduced system error. Instead of using a calibration marker, we adopted the existing, standardized features on the robot flange as the reference for the depth sensor. The calibration accuracy is found to be as high as the camera's, probing the hardware limit of the robot system. In particular, with the availability of Photoneo's MotionCam-3D camera, we can acquire quality point cloud data in high resolution, accuracy, and frame rate. This enables us to extend our proposed method to other geometric features on the robot, such as the wrist joint, for dynamic tracking of the calibration errors using the whole body of the robot. The limitation of this paper includes the camera used, which is a test model from a specific brand. A future research direction is to test the proposed method further using different depth sensors of similar capabilities. Another future direction is to extend the proposed method to other problems such as pose estimation or object tracking.

## Data Availability Statement

The datasets generated for this study are available on request to the corresponding author.

## Author Contributions

FW contributed 70% of the experiment setup, theoretical analysis, and paper writing. CS contributed 30% of the work, mainly involves the discussion of the experiment design and paper writing.

### Conflict of Interest

FW was employed by the company AncoraSpring, Inc. The remaining author declares that the research was conducted in the absence of any commercial or financial relationships that could be construed as a potential conflict of interest.
